# Kidney transplant outcomes in HIV-positive patients: a systematic review and meta-analysis

**DOI:** 10.1186/s12981-019-0253-z

**Published:** 2019-11-20

**Authors:** Xin Zheng, Lian Gong, Wenrui Xue, Song Zeng, Yue Xu, Yu Zhang, Xiaopeng Hu

**Affiliations:** 1 Urology Institute, Capital Medical University, Beijing, China; 2 Department of Urology, Beijing Chaoyang Hospital, Capital Medical University, 8 Gongti Nanlu, Chaoyang District, Beijing, China; 3 Department of Urology, Beijing You-An Hospital, Capital Medical University, Beijing, China

## Abstract

**Background:**

Kidney transplantation is now a viable alternative to dialysis in HIV-positive patients who achieve good immunovirological control with the currently available antiretroviral therapy regimens. This systematic review and meta-analysis investigate the published evidence of outcome and risk of kidney transplantation in HIV-positive patients following the PRISMA guidelines.

**Methods:**

Searches of PubMed, the Cochrane Library and EMBASE identified 27 cohort studies and 1670 case series evaluating the survival of HIV-positive kidney transplant patients published between July 2003 and May 2018. The regimens for induction, maintenance therapy and highly active antiretroviral therapy, acute rejection, patient and graft survival, CD4 count and infectious complications were recorded. We evaluated the patient survival and graft survival at 1 and 3 years respectively, acute rejection rate and also other infectious complications by using a random-effects analysis.

**Results:**

At 1 year, patient survival was 0.97 (95% CI 0.95; 0.98), graft survival was 0.91 (95% CI 0.88; 0.94), acute rejection was 0.33 (95% CI 0.28; 0.38), and infectious complications was 0.41 (95% CI 0.34; 0.50), and at 3 years, patient survival was 0.94 (95% CI 0.90; 0.97) and graft survival was 0.81 (95% CI 0.74; 0.87).

**Conclusions:**

With careful selection and evaluation, kidney transplantation can be performed with good outcomes in HIV-positive patients.

## Background

Traditionally, human immunodeficiency virus (HIV)-positive patients (HIV+) has not been considered to be good candidates for solid-organ transplantation for the poor prognosis of HIV patients. However, with the introduction of antiretroviral combination therapy (cART), the survival of HIV+ patients have been great improved. While the frequency of Acquired Immune Deficiency Syndrome (AIDS)-related events has consequently decreased, mortality due to organ failure has become a significant concern.

The initial attempts at kidney transplantation (KT) in HIV+ patients led to poor outcomes, but better results occurred with the availability of highly active antiretroviral therapy (HAART) [1, 2].

In this scenario, KT started to be proposed as a treatment even as “standard-of-care” for end-stage renal disease (ESRD) in selected HIV+ patients [3].

A multicentre study in the USA found that the survival rates for HIV+ recipients fall between those reported for older KT recipients and for all recipients in the American national database [4].

Despite these encouraging results, many issues still need to be addressed. Among the more relevant are the elevated incidence of acute rejection (AR), lower patient survival (PS) and graft survival (GS), and the hurdles caused by the interaction of immunosuppressive and antiretroviral (ARV) drugs. We conducted a systemic review and meta-analysis to determine the effectiveness of KT in the presence of HIV. Specifically, we examined PS and GS, AR and infectious complications in HIV+ patients who have undergone KT.

## Methods

### Study design

The study design of a systematic review and meta-analysis was chosen to define the published evidence of the effectiveness of KT in HIV+ patients. The study followed the Preferred Reporting Items for Systematic Reviews and Meta-Analysis (PRISMA) statement standards [5]. Our review was registered at the International Prospective Register of Systematic Reviews (PROSPERO CRD42018109178).

### Search strategy

We searched the Medline (1966 to June 2018), EMBASE (1974 to June 2018), and Cochrane Controlled Trials Register databases to identify studies that referred to KT in HIV+ patients; we also searched the reference lists of the retrieved studies. The following search terms were used: KT, HIV+, AIDS. A combination of subject headings and keywords for KT, HAART, HIV+ recipient, allograft survival, antiretroviral therapy, donor selection, ESRD and immunosuppression was used for the literature search.

### Eligibility criteria

Cohort studies and case–control studies were all eligible for inclusion if they reported outcomes of KT in HIV+ patients. Studies reporting outcomes shorter than 12 months post transplantation and transplantation occurring before HAART were introduced were excluded. Articles were independently assessed by 2 reviewers (X Z and WR X) according to the predetermined eligibility criteria. Any disagreement between reviewers was resolved by discussion with a third reviewer (XP H).

### Data extraction

All data were extracted independently by 2 reviewers (X Z and WR X) onto a Microsoft Excel spreadsheet (XP Professional Edition; Microsoft Corp, Redmond, WA), and any discrepancies were resolved by consensus. The following information was collected for each study: the study country, sample size, inclusion criteria, exclusion criteria, induction and maintenance immunosuppression, HAART regimen, mean CD4 T-cell counts (CD4 counts) pre-transplant and post-transplant, infectious complications, post-transplant neoplasia, PS and GS at 1 and 3 years, and AR rate. In order to analyse data of Infectious complications (IC), all infections requiring hospitalization were registered.

### Quality grading of studies

The quality of each study used for the meta-analysis was assessed based on the Newcastle–Ottawa-Scale (NOS) for cohort studies [6]. The evaluation of study quality included the following three categories: (i) selection (4 items), (ii) comparability (2 items), and (iii) the assessment of outcome (3 items). The NOS ranges from zero to a maximum of 9 points. Five authors (X Z, W X, S Z, Y X and Y Z) independently assessed the articles. The overall NOS score was determined as the median of all 5 individual NOS assessments. Study quality was graded as good (≥ 8 points), fair (6 or 7 points), and poor (≤ 5 points) [6].

### Data analysis

We undertook the descriptive analyses to identify the number of studies with relevant data, the countries where the studies were conducted, and other population attributes. The transplant outcome data were pooled using different transformations according to their different normal distribution conditions.

The data for PS, GS, AR, IC at 1 year and PS at 3 years were analysed using log transformation.

The data for GS at 3 years were analysed using arcsine transformation.

The transformed data were combined to estimate the pooled percentages with 95% confidence intervals using a random-effects model.

The transformed data were combined to estimate the pooled percentages with 95% confidence intervals using a random effects model [7] and presented as forest plots. We assessed the heterogeneity among studies using the Cochran Q test ($\chi^{ 2}_{{{\text{n}} - 1}}$; p < 0.05 to denote statistical significance) and estimated the amount of variation by I^2^ [8]. Statistical sources of heterogeneity were explored by examining the relationship between one or more study-level characteristics and the effect sizes that were observed in the studies using weighted least squares meta-regression. A rank correlation test of funnel plot asymmetry (z) was used to assess the presence of publication bias. Statistical analysis was performed using the R statistical software package (R Development Core Team, Vienna, Austria; URL: http://www.R-project.org; version 2.9.0), using the software libraries ‘meta’ and ‘metafor’, for the meta-analysis and meta-regression models, respectively.

## Results

### Systematic study review

**Fig. 1:**
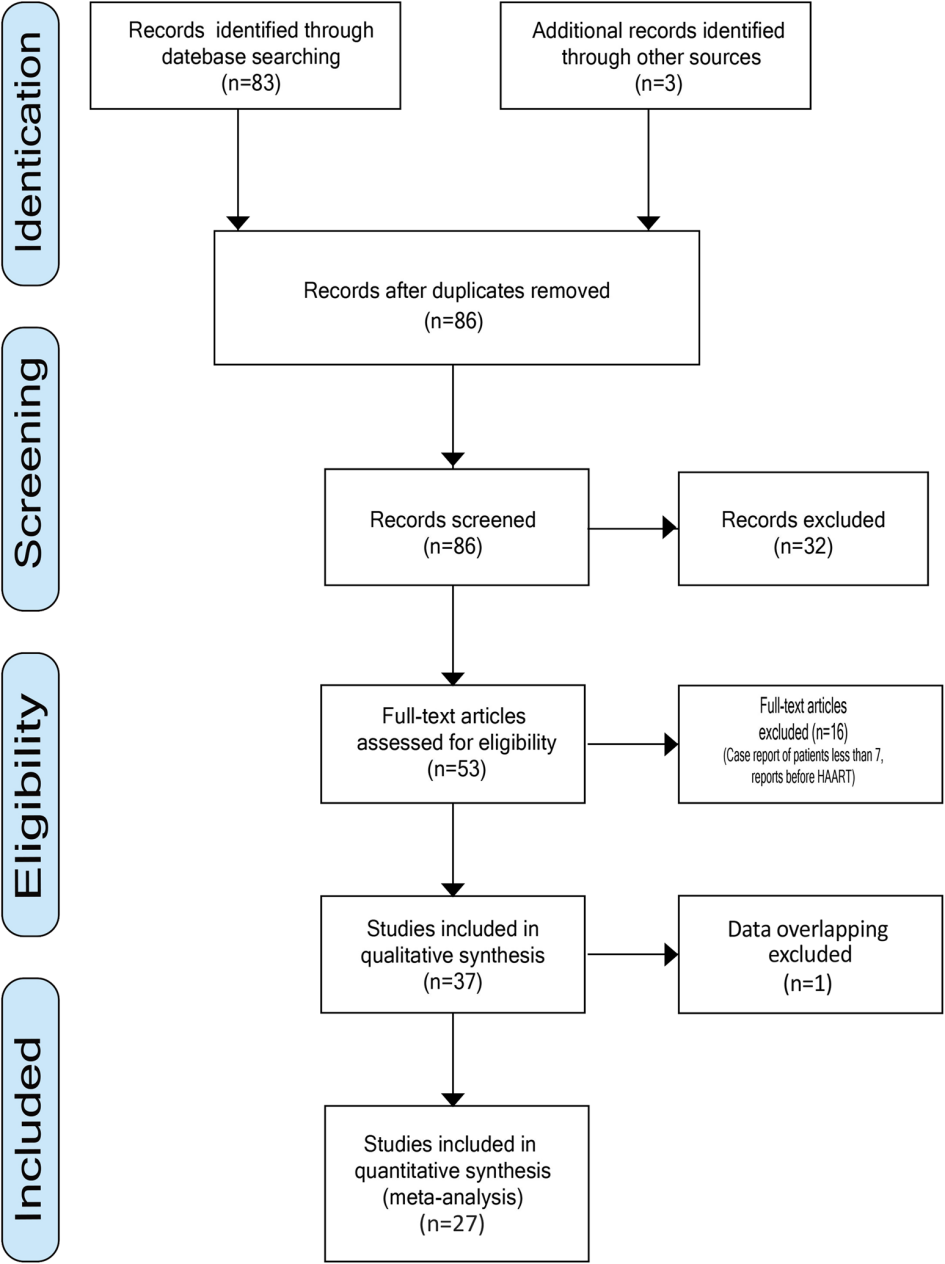
PRISMA flow chart of literature research

The search strategy identified 86 citations (Fig. 1), and among these, we identified 53 studies that appeared to bge relevant to our study. Finally, 27 of these studies, containing 1670 cases, met the inclusion criteria. Agreement between reviewers for assessment of study eligibility was 100%.

Detailed characteristics of all included studies are provided in Table 1. A majority of the studies were conducted in the US or Europe. All these details are summarized in Tables 2 and 3.

**Table 1:** Identified studies for systematic review according to PRISMA guidelines

Study	Country	Sample size	Inclusion criteria	Exclusion criteria	The duration of dialysis	Duration of HIV infection	Study tipe
Roland (2008)	USA	18	Undetectable HIV for 3 months, CD4 T-cell counts ≥ 200/μL, No history of OI	Patients with previously treated opportunistic complications (except progressive multifocal leukoencephalopathy, chronic cryptosporidiosis, lymphoma and visceral Kaposi’s sarcoma [KS]) were eligible	Not specified	Not specified	Prospective study
Touzot (2010)	Paris	27	Not specified	Not specified	Not specified	Not specified	Retrospective cohort study
Mazuecos (2006)	Spain	10	CD4 T-cell counts ≥ 200/μL for more than 6 months, Undetectable HIV for 3 months, stable ART (in case of indicated) for longer than 3 months, and no presence of definite AIDS complications	History of AIDS-defining infection	7.6 + 6.6 (1–22) years	10.6 + 6.9 (2–19) years	Retrospective cohort study
Stock (2003)	USA	10	Undetectable HIV for 3 months; CD4 T-cell counts ≥ 200/μL; no history of opportunistic infections; and tolerating a stable ARV regimen for 3 months before transplant	AIDS-defining opportunistic infection; history of cancer or opportunistic neoplasm (except for treated basal cell carcinoma or in situ anogenital cancer), and HCV positivity in kidney patients with findings of cirrhosis on liver biopsy	Not specified	Not specified	Prospective study
Stock (2010)	USA	150	CD4 T-cell counts ≥ 200/μL and Undetectable HIV for while receiving stable ART in the 16 weeks before transplantation	Patients with previously treated opportunistic complications, with the exception of progressive multifocal leukoencephalopathy, chronic intestinal cryptosporidiosis, primary central nervous system lymphoma, and visceral Kaposi’s sarcoma	Not specified	Not specified	Prospective study
Kumar (2004)	USA	40	Patients be adherent to dialysis treatment and HAART, have plasma HIV-1 RNA < 400 copies/mL, and absolute CD4 T-cell counts ≥ 200/μL	Not specified	Not specified	Not specified	Retrospective cohort study
Qiu (2006)	USA	38	Not specified	Not specified	Not specified	Not specified	Registry study
Tan (2004)	USA	7	Undetectable HIV for 3 months, CD4 T-cell counts ≥ 200/μL	Not specified	Not specified	Not specified	Retrospective cohort study
Carter (2006)	USA	20	First, candidates met standard criteria for placement on the kidney transplant waiting list. Second, candidates had undetectable HIV for 3 months, CD4 T-cell counts ≥ 200/μL for 6 months	History of cancer or opportunistic neoplasm (except for treated basal cell carcinoma, cutaneous Kaposi’s sarcoma or in situ anogenital cancer), prior transplant, pregnancy, significant HIV-related wasting (> 5% weight loss over 3 months), coinfection with hepatitis C with evidence of cirrhosis on liver biopsy, history of chronic intestinal cryptosporidiosis of > 1 month duration, history of progressive multifocal leukoencephalopathy or documented resistant fungal infections	Not specified	Not specified	Prospective study
Gruber (2008)	USA	8	(1) CD4 T-cell counts ≥ 200/μL and ultrasensitive viral load (USVL) less than 50 RNA copies/mL for more than or equal to 6 months and (2) no history of significant AIDS-associated opportunistic infections or neoplasms, both while on highly active antiretroviral therapy (HAART)	Not specified	Not specified	Not specified	Retrospective cohort study
Gómez (2013)	Spain	7	Patients do not suffer from any condition; CD4 T-cell counts ≥ 200/μL; Undetectable viral load (< 50 copies/mL); Social stability; Adherence to treatment In drug abusers: period of abstinence of at least 2 years	Not specified	Not specified	Not specified	Retrospective cohort study
Izzo (2017)	Italy	28	CD4 T-cell counts ≥ 200/μL, undetectable HIV RNA (if the patient was on cART) and presumable good compliance to follow up and therapy	Not specified	Not specified	Not specified	Retrospective cohort study
Roland (2004)	USA	26	CD4 T-cell counts ≥ 200/μL; undetectable HIV RNA	Elevated HIV RNA Level, Low CD4 T-Cell Count, History Of Opportunistic Infection Or Neoplasm, Or Incompletely Evaluated Altered Mental Status	Not specified	Not specified	Retrospective cohort study
Gasser (2009)	USA	27	Undetectable plasma HIV RNA for 6 months before transplantation, CD4 T-cell counts ≥ 200/μL and no use of IL-2 or GM-CSF in the 6 months prior to transplantation	Pregnancy and significant wasting or weight loss	Not specified	Not specified	Prospective study
Gathogo (2014)	UK	35	CD4 T-cell counts ≥ 200/μL and undetectable HIV RNA levels for a minimum of 6 months	Not specified	4.2 years	7.2 years	Retrospective cohort study
Baisi (2016)	Italy	18	Patients never treated with ARVs with CD4 T-cell counts ≥ 200/μL Patients on ARVs with CD4 T-cell counts ≥ 200/μL stable for at least 12 months and plasma HIV-RNA undetectable at the time of inclusion on waiting list Compliance to/willingness to continue ARVs and prophylaxis of opportunistic infections, if indicated If female, pregnancy test (b-HCG) negative (monthly monitoring)	History of AIDS-defining opportunistic infections in the previous 2 years History of neoplasm (with the exception of in situ cervical neoplasia and baso-cellular carcinoma with a documented disease-free period of more than 5 years; recovery from malignant disease must be certified by an oncologist) Detectable peripheral blood HHV DNA VL Breast-feeding underway	Not specified	Not specified	retrospective cohort study
Xia (2014)	USA	243	Not specified	Exclusions were multi-organ transplants and recipients that were pediatric, hepatitis B surface antigen positive, had missing or unknown HIV or HCV serostatus or received a previous liver transplant. Additional exclusions were recipient HIV-seropositivity and donor hepatitis C seropositivity	83.5% of patients Pretransplant dialysis > 3 years	Not specified	Registry study
Locke (2015)	USA	481	Not specified	Not specified	Not specified	Not specified	Registry study
Abbott (2004)	USA	47	Not specified	Not specified	4.8 ± 5.0 years	Not specified	
Cristelli (2017) Brazil	Brazil	39	Not specified	Not specified	42 months	96 months	Retrospective cohort study
Cristelli (2017) Spain	Brazil	15	Not specified	Not specified	84 months	120 months	Retrospective cohort study
Mazuecos (2013)	Spain	36	a. CD4 T-cell counts ≥ 200/μL for > 6 months b. HIV-1 RNA undetectable c. On stable anti-retroviral therapy > 3 months d. No other complications from AIDS (e.g., opportunistic infection, including aspergillus, tuberculosis, coccidioide-mycosis, resistant fungal infections, Kaposi’s sarcoma or other neoplasm) e. Meeting all other criteria for kidney transplantation	1. Metastatic cancer 2. Ongoing or recurring infections that are not effectively treated 3. Serious cardiac or other ongoing insufficiencies that create an inability to tolerate transplant surgery 4. Serious conditions that are unlikely to be improved by transplantation as life expectancy can be finitely measured 5. Demonstrated patient noncompliance, which places the organ at risk by not adhering to medical recommendations 6. Potential complications from immunosuppressive medications are unacceptable to the patient (e.g., the benefits of staying on dialysis outweigh the risks associated with transplantation) 7. AIDS (diagnosis based on CDC definition of CD4 T-cell count < 200/μL)	49.5 months	Not specified	Retrospective cohort study
Rosa (2016)	USA	58	Not specified	Not specified	Not specified	Not specified	
Vicari (2016)	Brazil	53	Being clinically stable under HAART, having at least a 6-month period of stable CD4 T-cell counts ≥ 200/μL, and undetectable viral load	Not specified	Not specified	Not specified	Prospective study
Bossini (2014)	Italy	13	CD4 T-cell counts ≥ 200/μL and undetectable plasma HIV type-1 RNA levels based on an ultrasensitive polymerase chain reaction assay while receiving stable HAART during the 3 months before transplantation	History of progressive multifocal leukoencephalopathy, chronic intestinal cryptosporidiosis, lymphoma, or visceral Kaposi’s sarcoma	5.0 ± 3.1 years	Not specified	Registry study
Mazuecos (2011)	Spain	20	a. CD4 T-cell counts ≥ 200/μL for > 6 months b. HIV-1 RNA undetectable c. On stable anti-retroviral therapy > 3 months d. No other complications from AIDS (e.g., opportunistic infection, including aspergillus, tuberculosis, coccidioide-mycosis, resistant fungal infections, Kaposi’s sarcoma or other neoplasm) e. Meeting all other criteria for kidney transplantation	1. Metastatic cancer 2. Ongoing or recurring infections that are not effectively treated 3. Serious cardiac or other ongoing insufficiencies that create an inability to tolerate transplant surgery 4. Serious conditions that are unlikely to be improved by transplantation as life expectancy can be finitely measured 5. Demonstrated patient noncompliance, which places the organ at risk by not adhering to medical recommendations 6. Potential complications from immunosuppressive medications are unacceptable to the patient (e.g., the benefits of staying on dialysis outweigh the risks associated with transplantation) 7. AIDS (diagnosis based on CDC definition of CD4 count < 200 cells/mm^3^)	6.53 ± 5.62 years	8.45 ± 5.01 years	Prospective study
Gathogo (2016)	UK	76	Not specified	Not specified	4.9 years	Not specified	Registry study
Malat (2018)	USA	120	An undetectable viral load, CD4 T-cell counts ≥ 200/μL, and be on an ART regimen for at least 6 months	Not specified	16 years	Not specified	Retrospective cohort study

The paper by Cristelli et al. contains two cohorts from Brazil and Spain separately, so we treat it as two independent cohorts

**Table 2:** Immunosuppression and rejection

Study	Follow-up days [mean ± SD or median (range)]	Induction	Maintenance	Type of rejection	Treatment of rejection
Roland 2008)	1520 ± 593 days	Anti-CD25	CSA, Steroids ± MMF	Acute cellular 14 (78%) Acute vascular 1 (6%) Acute cellular and vascular 2 (11%)	Not specified
Touzot (2010)	29 months (range 12–48 months)	Antiinterleukin 2 receptor antibody (Basiliximab, Novartis, 20 mg at day 0 and day 4) (26) and polyclonal antithymocyte globulins (1) (Thymoglobuline, Genzyme, 1.5 mg/kg/day during 4 days)	CSA or D29, Steroids ± MMF. MMF was given at 1000 mg twice a day. Methylprednisolone was given as followed: 500 mg intravenously at day 0 and 125 mg at day 1. From day 2, 20 mg/day of oral prednisone was given and tapered progressively to 10 mg/day at 6 months and 5 mg/day at 9 months	Acute cellular rejection	Steroid pulses
Mazuecos (2006)	489 ± 468 days	ATG(1); Anti-CD25(3)	TAC, MMF and steroids	Not specified	Mpred (250 mg) Rituximab (for AMR)
Stock (2003)	480 ± 300 days	Not used	CSA, MMF and steroids	Not specified	Mild rejection was treated with bolus steroids and a switch in maintenance immunosuppression from CSA to tacrolimus. Vascular (type II) rejection was treated with the polyclonal anti-T-cell agent Thymoglobulin, bolus steroids, and a switch in maintenance immunosuppression from + D15 to tacrolimus
Stock (2010)	1.7 years	An induction therapy by a monoclonal antiinterleukin 2 receptor antibody, antithymocyte globulin (ATG), or both was permitted	Initial immunosuppressive therapy included glucocorticoids, CSA or TAC, and MMF. Sirolimus was used in patients with calcineurin-inhibitor-associated nephrotoxicity	Acute cellular rejection episodes(42) Acute vascular rejection episodes(4) Acute cellular and vascular rejection episodes combined(7) Chronic and acute rejection episodes(4)	Not specified
Kumar (2004)	730 days	Antiinterleukin 2 receptor antibody	Cyclosporine, sirolimus, and Steroids.	Cell and antibody mediated rejection (2/9)	Methylprednisolone(9) Intravenous immune globulin and rituximab(2)
Qiu (2006)	1825 days	Anti-CD25 (23)	CSA(20); Tac(13); Sir(14); Steroid-sparing(1)	Not specified	Not specified
Tan (2004)	1485 ± 425 days; 246 ± 87 days	None (42%) (deceased donor) Alemtuzumab (57%) (living-related donor)	TAC, MMF and Steroids	Not specified	Not specified
Carter (2006)	854 days	Induction therapy with lymphocyte-depleting agents was avoided. IL-2 receptor inhibitor induction was used	All patients received perioperative steroids, MMF (2–3 g/day), a calcineurin inhibitor (either cyclosporine or TAC), and/or sirolimus		Treatment for acute rejection consisted of 3 days of high-dose methylprednisolone, followed by a prednisone taper, and increased maintenance immunosuppression, which frequently meant switching the recipient from cyclosporine to tacrolimus. Additionally, moderate-to-severe cases of rejection were treated with thymoglobulin on an individualized basis
Gruber (2008)	15 months	All patients received induction therapy with antiinterleukin 2 receptor antibody (basiliximab 20 mg on postoperative days 0 and 4) or daclizumab (1.5 mg/kg on days 0 and 7)	CSA, MMF and Steroids	Not specified	Borderline or grade I rejection episodes were treated with methylprednisolone 500 mg IV for 3 days, followed by a steroid taper. Steroid-resistant grade I, and grade II rejections were treated with 5 to 7 daily doses of Thymoglobulin with target absolute CD3 counts less than or equal to 10
Gómez (2013)	16.0 months (range 3.0 to 96.6 months)	Iinduction therapy used antiinterleukin 2 receptor antibody (baxiliximab) (3/7)	TAC, MMF and Steroids	Not specified	Patients were treated with steroid pulses, which reversed acute rejection and improved renal function
Izzo (2017)	126.1 weeks	The patients received an induction therapy with antiinterleukin 2 receptor antibody (basiliximab) in two doses. Intravenous methylprednisolone was given in tapering doses and discontinued on day 5 after transplantation,or received basiliximab, methylprednisolone and antilymphocyte serum as induction therapy	TAC, MMF and Steroids	Not specified	Not specified
Roland (2004)	314 days (3–1696)	Not specified	CSA, MMF and Steroids	Not specified	Not specified
Gasser (2009)	Not specified	Ten of the 27 transplant recipients received antithymocyte globulin (ATG) perioperatively (i.e. immediately prior to transplantation [n = 9], or within the first 12 weeks posttransplantation [n = 1])	Twenty-five of the 27 [92.6%] individuals were initiated on a standard triple IS regimen consisting of steroids (Prednisone), a calcineurin inhibitor (Cyclosporine A or TAC) and a nucleotide/DNA synthesis inhibitor (MMF or Azathioprine)	Not specified	Not specified
Gathogo (2014)	Not specified	Of the 32 patients with available data, 30 (88%) received induction immunosuppressive therapy consisting of basiliximab (73%) or daclizumab (27%) with methylprednisolone, and two patients received methylprednisolone only. 30 (88%) received induction immunosuppressive therapy consisting of basiliximab (73%) or daclizumab (27%) with methylprednisolone, and two patients received methylprednisolone only	All patients received triple maintenance immunosuppressive therapy consisting of a CNI, mycophenolate or azathioprine, and Steroids	Not specified	Six patients responded to pulsed corticosteroid; other or additional treatment interventions to combat AR included intravenous immunoglobulin (IVIG, 1⁄44), plasma exchange (1/41), ATG (1/41), rituximab (1/42) and augmentation of baseline immunosuppression (1/48)
Baisi (2016)	3.1 years	Two recipients received induction therapy with a standard dose of basiliximab; 500 mg intravenous (IV) methylprednisolone (MP) was given intra-operatively, followed by oral prednisolone progressively tapered from 16 mg to complete withdrawal within the 3rd month	Immunosuppression protocol included a delayed CSA (2.5 mg/kg bid when creatinine was < 3.0 mg/dL) targeted to maintain CSA (C2 level) at initial value of 1000 ng/mL. At post-operative day (pod) 21, everolimus (EVL) 0.75 mg bid was introduced (EVL 0.75 mg bid; target EVL trough blood levels [TLC]: 8e10 ng/mL and CsAC2: 400e500 ng/mL); steroid was tapered to 4 mg/day within 45 days. After 6 months, EVL and CsA blood levels were targeted to EVLTLC 6 to 8 ng/mL and CsAC2, 250 to 350 ng/mL. After the first 6 case, mycophenolic acid (MPA) 720 mg bid was added until pod 21	Not specified	Not specified
Xia (2014)	Not specified	Not specified	Not specified	Not specified	Not specified
Locke (2015)	3.8 years	Not specified	Not specified	Not specified	Not specified
Abbott (2004)	2.62 ± 1.32 years	Induction antibody use(22)	Cyclosporine(30) TAC(19) MMF(38) AZA(7)	Not specified	Not specified
Cristelli (2017) Brazil	2.8 years ± 2.51	No induction(17) ATG(11) Antiinterleukin 2 receptor antibody (basiliximab)(11)	TAC, MMF and Steroids(23) CSA, MMF and Steroids(2) TAC, AZA and Steroids(12) Other(2)	Borderline changes(5), IA(6), IB(7), IIA(1), IIB(3)	Not specified
Cristelli (2017) Spain	4.6 years ± 2.85	No induction(2) ATG(6) antiinterleukin 2 receptor antibody (basiliximab)(7)	TAC, MMF and Steroids(12) MTOR,MMF and SteroidsF(3)	Borderline changes(2), IA(1), IB(0), IIA(1)	Not specified
Mazuecos (2013)	33.6 months	Not specified	Not specified	Borderline/IA(3), IB(2), IIA(4), Antibody-mediated(2)	Not specified
Rosa (2016)	1028 ± 813 days	All of the patients received anti–thymocyte globulin, basiliximab and methylprednisolone for induction.	Prednisone(52), IVIG(5), Rituximab(7), TAC(57), MMF(57), Sirolimus(3), Cyclosporine(2)	Not specified	Not specified
Vicari (2016)	Not specified	No induction(26) ATG(5) antiinterleukin 2 receptor antibody (basiliximab)(22)	Steroids(53), TAC(40), Cyclosporine(10), MMF(41), AZA(9), mTOR inhibitors(1)	Antibody-mediated AR(2) Antibody-mediated AR(3)	Not specified
Bossini (2014)	50 ± 22.0 months	Antiinterleukin 2 receptor antibody (basiliximab) and methylprednisolone	TAC or cyclosporine and MMF	CMR(4), AMR(4), and both CMR and AMR (mixed)(4). Overall, indicators of AMR were present in eight of 12 episodes (66.6%)	Acute cellular-mediated rejections (CMR) were treated with methylprednisolone (MP) at high doses (800–1000 mg divided into 4 days) and subsequently tapered to a daily dose between 8 and 4 mg/day to be maintained indefinitely. Treatment of antibody-mediated rejection (AMR) involved a combination of multiple modalities, including high doses of steroids, plasma exchange, intravenous immunoglobulins (IVIg), and thymoglobulin
Mazuecos (2011)	39.98 ± 36.51 months	Anti-CD25(6), Thymoglobulin(1)	TAC(18) MMF(2) Mycophenolate(20)	Antibody mediated acute rejection	
Gathogo (2016)	Not specified	Antiinterleukin 2 receptor antibody (basiliximab)(68) Alemtuzumab(2) Rituximab + plasma exchange(1) Pulsed corticosteroids only(2)	Calcineurin inhibitor + MMF or AZA +Steroids(76) TAC monotherapy(2)	Not specified	Not specified
Malat (2018)	16 years	Antiinterleukin 2 receptor antibody (basiliximab)	Calcineurin inhibitors (CNIs), sirolimus, and Steroids TAC, MMF, and low-dose Steriods Belatacept(3)	Not specified	Not specified

**Table 3:** HIV and related complications

Study	HAART regimen	Pharmacokinetic changes	Mean CD4 T-cell counts pre-TX (cells/μL)	Mean CD4 T-cell counts post-TX	Prophylaxis against opportunistic infection	Infectious complications	Post-transplant neoplasia
Roland (2008)	Varied (zidovudine and stavudine avoided)	Not specified	439 (293–613)	Not specified	OI prophylaxis included life-long trimethoprim-sulfamethoxazole, dapsone or atovaquone to prevent *Pneumocystis carinii* pneumonia (PCP), brief antifungal prophylaxis using fluconazole, and *Cytomegalovirus* (CMV) prophylaxis with either acyclovir or valcyte, depending upon the recipient and donor CMV status	Candida esophagitis(1); CMV(1)	Not specified
Touzot (2010)	Not specified	Because of persistent high trough level of CNI, protease inhibitor treatment was stopped in nine patients during the first week of posttransplantation and in five others during the follow-up	386	545 (3 months) 534 (6 months) 460 (12 months) 569 (24 months)	Patients received ganciclovir or valgangyclovir for cytomegalovirus and trimethoprim/sulfamethoxazole for *Pneumocystis jirovecii*, for at least 6 months. For patients with a past history of tuberculosis, Isoniazid was added for 9 months	Pyelonephritis(18) Pneumonia(5) Septic shock(1) Others(4) CMV(2) BK virus(1)	Lymphoma(1)
Mazuecos (2006)	Varied	Not specified	≥ 200	670 ± 481	Not specified	Pneumonia(3) VZV(1)	Not specified
Stock (2003)	Varied	Not specified	423 ± 93	419 ± 287	Standard prophylaxis for Pneumocystis, cytomegalovirus (CMV), and fungal infections were used according to standard transplant protocols	*Staphylococcus aureus* wound infection(2) Haemophilus influenza bacterial pneumonia(1) *S. aureus* endocarditis(1)	Not specified
Stock (2010)	Protease-inhibitor–based(63) NNRTI-based(59) Protease-inhibitor-based and NNRTI-based(15) Nucleoside analogues only(5) Nucleoside analogues only(6) None(2)	Not specified	524	Not specified	Prophylaxis against opportunistic infection included lifelong therapy to prevent *Pneumocystis jirovecii* pneumonia, fluconazole for antifungal prophylaxis, and valganciclovir or ganciclovir to prevent cytomegalovirus infection. Macrolide prophylaxis against Mycobacterium avium complex was required when the CD4+ T-cell count dropped below 75 cells per cubic millimeter	*Pseudomonas aeruginosa* sepsis(1)	Renal-cell carcinoma(2) Kaposi’s sarcoma(2) Oral squamous-cell carcinoma(2) Squamous-cell skin cancer(1) Basal-cell skin cancer(1) Thyroid gland cancer(1)
Kumar (2004)	Varied	All patients continued their HAART regimens.	≥ 200	≥ 400	Infection prophylaxis was ganciclovir or valgancyclovir for cytomegalovirus, trimethoprim/sulfamethoxazole or dapsone for *Pneumocystis carinii*, and nystatin for oral and esophageal thrush for 200 days after transplantation	Sepsis(1) Chest infection(2) Necrotizing fasciitis(1) Infection of lymphocoele(1) Admitted urinary tract infection(9)	Not specified
Qiu (2006)	Not specified	Not specified	Not specified	Not specified	Not specified	Bacterial pneumonia(1)	Not specified
Tan (2004)	Varied	Not specified	589 ± 313 946 ± 800	424 ± 384	Not specified	Plantar fasciitis(1)	Basal cell carcinoma
Carter (2006)	Not specified	Patients resumed their pre-transplant HAART therapy when an oral diet was started, typically 1 or 2 days after transplant.	Not specified	Not specified	Varied	Candida oesophagitis(1) *S. aureus* endocarditis with septic embolization(1) *Streptococcus viridans* bacteraemia(1) *Pseudomonas pneumonia* with multi-organ failure(1) *Escherichia coli* urosepsis(1) Culture-negative urosepsis(1) Enterococcus bacteraemia(1) *Polymicrobial pneumonia* sepsis(1) *Clostridium difficile* colitis(1) Diverticulitis and secondary bacterial peritonitis(1) Influenza, bacterial pneumonia(1) *Pseudomonas pneumonia*(1)	Not specified
Gruber (2008)	All recipients were maintained on at least two nucleoside reverse transcriptase inhibitors, three in combination with a ritonavir-boosted protease inhibitor (PI), two in combination with a non-boosted PI, and two in combination with nevirapine (a nonnucleoside reverse transcriptase inhibitor)	Not specified	≥ 200	≥ 200	Antimicrobial prophylaxis was initiated within the first 24 to 48 h after surgery. All patients received trimethoprim/sulfamethoxazole one single-strength daily for 6 months and nystatin 5 mL four times per day for 1 month. Cytomegalovirus prophylaxis was administered depending on the patient’s risk-stratified profile	CMV(1) Pneumonia(1) Urinary tract infection(3)	Not specified
Gómez (2013)	Not specified	Protease inhibitor treatment was stopped with substitution of the integrase inhibitor Raltegravir	504	373.5 (3 months) 488 (6 months)	Patients received trimethoprim–sulfamethoxazole for *Pneumocystis jiroveccii*	Not specified	Epstein–Barr virus high grade-related B-cell lymphoma(1)
Izzo (2017)	Not specified	To avoid PK interactions, cART was modified from a PI/ NNRTI-based to an InSTI-based regimen in 11/20 patients alive with functioning graft (65%); 7/11 were switched to raltegravir and 4/11 were switched to dolutegravir. 7/20 (35%) were on treatment with a cART regimen including both InSTI and PI/ritonavir (RTV) or efavirenz (EFV) at the end of follow-up	337	400	Not specified	Pneumonia and urinary tract infections were the most common diagnosis	Skin Kaposi’s sarcoma(2) Colorectal cancer(1)
Roland (2004)			441 (200–1054)	436 (3–975)	Not specified	*Candida esophagitis*(1); *Staphylococcal sepsis*(1)	Not specified
Gasser (2009)	ART consisted of nucleoside/nucleotide reverse transcriptase inhibitors (RTI) and/or non-nucleoside RTI and/or protease inhibitors, mostly combined as a three-class therapy	Not specified	483	Not specified	Not specified	Not specified	Not specified
Gathogo (2014)	Antiretroviral therapy was stratified as containing ritonavir-boosted protease inhibitors, non-nucleoside reverse transcriptase inhibitors (NNRTI) or other (regimens containing nucleoside/nucleotide reverse transcriptase with or without integrase inhibitors)	Not specified	366	Not specified	Regarding the management and prevention of cytomegalovirus (CMV) infection, some centres routinely administered valganciclovir prophylaxis for 3 months posttransplantation (irrespective of donor/recipient CMV IgG status), while others prescribed CMV prophylaxis to recipients of grafts from CMV IgG-positive donors or combined regular posttransplant CMV surveillance with preemptive valganciclovir treatment if the CMV viral load exceeded 3–4000 copies/mL	Urinary tract infection(10) Pneumonia(5) Cellulitis(2) Pyrexia of unknown origin(1) Herpes simplex viral encephalitis(1)	Not specified
Baisi (2016)	To avoid drug interactions between protease inhibitors and IS, ARV was given in the immediate post-operative period with enfuvirtide in combination with 2 nucleoside analogues or 1 nucleoside analogue and raltegravir (RAL), which was administered within 48 h	Once steady state of IS was achieved (on average, pod 30), T20 was stopped and HAART was modified on the basis of HIV pre-transplant genotype profile, individual drug tolerability, and clinical conditions	441	Not specified	For *Pneumocystis jirovecii* prophylaxis, we used a 6 month course of trimethoprim–sulfametoxazol. For CMV prophylaxis, all patients received IV ganciclovir or oral valganciclovir for a 3-month treatment; in the case of donor/recipient CMV status, specific anti-CMV immunoglobulins were added	Not specified	No neoplasms were reported
Xia (2014)	Not specified	Not specified	Not specified	Not specified	Not specified	Not specified	Not specified
Locke (2015)	Not specified	Not specified	Not specified	Not specified	Not specified	Not specified	Not specified
Abbott (2004)	Not specified	Not specified	Not specified	Not specified	Not specified	Not specified	Not specified
Cristelli (2017) Brazil	Non-boosted protease-inhibitor(2) Boosted protease inhibitor(16) NNRTI(20) Nucleoside analogs only(2)	Need for antiretroviral changes(14) Drug interactions with CNI/mTORi(3) Therapeutic failure(5) Adverse events(4) Unavailable drug(1) Unclear reason(1)	> 200	356 (3 months) 502 (1 year) 556 (3 years)	All patients received prophylactic trimethoprim/sulfa-methoxazole against *Pneumocystis jirovecii* and toxoplasmosis for at least 6 months	Surgical site(5) Urinary tract(13) Respiratory tract(15) Cytomegalovirus(7) Varicella-zoster virus(6) Esophageal candidiasis(5)	Non-skin cancer(1)
Cristelli (2017) Spain	Non-boosted protease-inhibitor(2) Boosted protease inhibitor(5) NNRTI(4) Integrase inhibitor(4)	Need for antiretroviral changes(9); Drug interactions with CNI/mTORi(8); Unclear reason(1)	> 200	403 (3 months) 491 (1 year) 456(3 years)	All patients received prophylactic trimethoprim/sulfamethoxazole against *Pneumocystis jirovecii* and toxoplasmosis for at least 6 months	Surgical site(1) Urinary tract(5) Respiratory tract(3) Cytomegalovirus(1)	Non-skin cancer(2)
Mazuecos (2013)	Not specified	A trend was observed to increase non-nucleoside reverse transcriptase inhibitors use, although without significant differences at the end of the study. Protease inhibitors continued to be administered after KT, but their use dropped significantly at the end. On the contrary, the use of integrase inhibitor (raltegravir) increased most significantly after KT, and that increase was maintained at the end of the study, suggesting a good tolerance to the drug	420	413 (1 month) 497 (3 months) 570 (1 year) 627 (2 years) 618 (3 years)	The main prophylactic therapies for infections included trimethoprim–sulfamethoxazole for Pneumocystis (at least 6 months), ganciclovir/valganciclovir for cytomegalovirus (at least 3 months) and isoniazid for patients with a past history of tuberculosis (9 months)	Bacterial infection(41) Fungal infection(2) Viral infection(6)	Skin carcinoma(3) Kaposi’s sarcoma(1) Lymphoproliferative disorder(1)
Rosa (2016)	The three most common regimens post-transplant were nucleoside reverse transcriptase inhibitors (NRTI) plus PI, NRTI plus INSTI, and NRTI plus NNRTI	A total of 30 (52%) patients underwent ART modifications after transplan	546.07 ± 271.04	318.54 ± 240.73 (12 months) 374.14 ± 235.68 (26 months) 401.57 ± 283.71 (52 months)	Not specified	CMV(11) Others not specified	Not specified
Vicari(2016)	Reverse transcriptase inhibitors were used for all patients, Non-nucleoside reverse transcriptase inhibitors were used by 29 patients, and protease inhibitors were used by 21 patients	Not specified	577.3 ± 333.5	610.3 ± 318.5	Not specified	Bacterial infection(55) Cytomegalovirus infection(39) Polyoma virus infection(7) Other viral infections(8) (Include herpes simplex, varicella zoster, adenovirus, and dengue)	Not specified
Bossini (2014)	The HAART regimen was protease inhibitor (PI)-based in 10 cases and non-nucleoside reverse transcriptase inhibitor (NNRTI)-based in the last two patients	Antiretroviral therapy was temporarily interrupted on the day of transplantation and restarted within 4 days. Only two patients remained without HAART after transplantation because they maintained an adequate immunological and virological control	352 ± 174	352 ± 174 (1 year)	Trimethoprim–sulfamethoxazole for 6 months	Pneumonia(5) HSV 2 genitalis(1) Malaria(1) CMV infectious(3) UTI(3) Epididymitis(2) Esophageal candidiasis(1) BKVN(1)	Kaposi’s sarcoma(1)
Mazuecos (2011)	Not specified	Two patients remained without HAART after transplantation because they maintained an adequate immunological andvirological control	> 200	> 200	Not specified	Bacterial(10) Mycotic(1) CMV(1) Other virus(2)	Lymphoma(1)
Gathogo (2016)	PI/r containing(30) NNRTI containing(40) Integrase inhibitor containing Raltegravir(23)	Not specified	366	Not specified	Not specified	Not specified	Not specified
Malat (2018)	Varied	Not specified	≥ 200	Not specified	Not specified	Not specified	Not specified

Regarding immunosuppression and rejection, most of the patients received antibody induction therapy with different regimens containing basiliximab, daclizumab, antithymocyte globulin (ATG) or methylprednisolone. The maintenance regimens were mainly composed of cyclosporin A (CSA), mycophenolate mofetil (MMF), tacrolimus (TAC) and steroids, which were the same as the maintenance regimens for HIV-negative patients.

Mean CD4 counts were steady in most of the patients. As we observed, in all the cohorts with available data (22/27 cohorts), mean CD4 counts pre-TX and post-TX were greater than 200 cells/μL, and even elevated post transplantation.

Prophylaxis against opportunistic infection was common in most studies as follows: patients received ganciclovir or valganciclovir for cytomegalovirus and trimethoprim/sulfamethoxazole, dapsone or atovaquone for *Pneumocystis jirovecii* for at least 6 months.

Kaposi’s sarcoma and skin cancer were the most observed post-transplant neoplasia. As we showed in Table 3, in all the cohorts with available data (11/27 cohorts, 360 cases in total), there are 6 cases of Kaposi’s sarcoma, 6 cases of skin cancer, 4 cases of lymphoma and 9 cases of other neoplasia.

### Quality of studies included in the meta-analysis

Each of the 27 studies included in the meta-analysis was assessed by the NOS to investigate the risk of bias within the studies. Table 4 shows the results of the quality assessment. None of the studies had less than three points in the category selection. Two studies controlled for age and gender, and 9 controlled for other factors, such as the HAART regimen and/or immunosuppression therapy. Finally, 5 studies were graded as good quality and 22 as fair quality.

**Table 4:** NOS score

Author (refs.)	Representativeness of the exposed cohort	Selection of the non-exposed cohort	Ascertainment of exposure	Outcome of interest not present at start	Comparability: age and sex	Comparability: other factors	Assessment of outcome	Follow-up long enough	Adequacy of follow-up	Total NOS score	Study quality
Roland [48]	1 ●	0 ○	1 ●	1 ●	0 ○	0 ○	1 ●	1 ●	1 ●	6	Fair
Touzot [31]	1 ●	0 ○	1 ●	1 ●	0 ○	0 ○	1 ●	1 ●	1 ●	6	Fair
Mazuecos [49]	1 ●	0 ○	1 ●	1 ●	0 ○	0 ○	1 ●	1 ●	1 ●	6	Fair
Stock [50]	1 ●	0 ○	1 ●	1 ●	0 ○	0 ○	1 ●	1 ●	1 ●	6	Fair
Stock [4]	1 ●	0 ○	1 ●	1 ●	0 ○	0 ○	1 ●	1 ●	1 ●	6	Fair
Kumar [51]	1 ●	0 ○	1 ●	1 ●	0 ○	0 ○	1 ●	1 ●	1 ●	6	Fair
Qiu [52]	1 ●	1 ●	1 ●	1 ●	0 ○	0 ○	1 ●	1 ●	1 ●	7	Fair
Tan [53]	1 ●	0 ○	1 ●	1 ●	0 ○	0 ○	1 ●	1 ●	1 ●	6	Fair
Carter [54]	1 ●	0 ○	1 ●	1 ●	0 ○	0 ○	1 ●	1 ●	1 ●	6	Fair
Gruber [55]	1 ●	0 ○	1 ●	1 ●	0 ○	0 ○	1 ●	1 ●	1 ●	6	Fair
Gómez [56]	1 ●	0 ○	1 ●	1 ●	0 ○	0 ○	1 ●	1 ●	1 ●	6	Fair
Izzo [17]	1 ●	0 ○	1 ●	1 ●	0 ○	0 ○	1 ●	1 ●	1 ●	6	Fair
Roland [57]	1 ●	0 ○	1 ●	1 ●	0 ○	0 ○	1 ●	1 ●	1 ●	6	Fair
Gasser [58]	1 ●	0 ○	1 ●	1 ●	0 ○	0 ○	1 ●	1 ●	1 ●	6	Fair
Gathogo [10]	1 ●	1 ●	1 ●	1 ●	0 ○	1 ●	1 ●	1 ●	1 ●	8	Good
Baisi [59]	1 ●	0 ○	1 ●	1 ●	0 ○	0 ○	1 ●	1 ●	1 ●	6	Fair
Xia [20]	1 ●	1 ●	1 ●	1 ●	1 ●	1 ●	1 ●	1 ●	1 ●	9	Good
Locke [11]	1 ●	0 ○	1 ●	1 ●	1 ●	1 ●	1 ●	1 ●	1 ●	8	Good
Abbott [2]	1 ●	1 ●	1 ●	1 ●	0 ○	0 ○	1 ●	1 ●	1 ●	7	Fair
Cristelli Brazil [60]	1 ●	0 ○	1 ●	1 ●	0 ○	0 ○	1 ●	1 ●	1 ●	6	Fair
Cristelli Spain [60]	1 ●	0 ○	1 ●	1 ●	0 ○	0 ○	1 ●	1 ●	1 ●	6	Fair
Mazuecos [61]	1 ●	1 ●	1 ●	1 ●	0 ○	1 ●	1 ●	1 ●	1 ●	8	Good
Rosa [40]	1 ●	0 ○	1 ●	1 ●	0 ○	1 ●	1 ●	1 ●	1 ●	7	Fair
Vicari [30]	1 ●	1 ●	1 ●	1 ●	0 ○	0 ○	1 ●	1 ●	1 ●	7	Fair
Bossini [27]	1 ●	0 ○	1 ●	1 ●	0 ○	0 ○	1 ●	1 ●	1 ●	6	Fair
Mazuecos [9]	1 ●	1 ●	1 ●	1 ●	0 ○	1 ●	1 ●	1 ●	1 ●	8	Good
Gathogo [34]	1 ●	0 ○	1 ●	1 ●	0 ○	1 ●	1 ●	1 ●	1 ●	7	Fair
Malat [62]	1 ●	0 ○	1 ●	1 ●	0 ○	1 ●	1 ●	1 ●	1 ●	7	Fair
Sum ●	29	8	29	29	3	9	29	29	29		
Sum **○**	0	21	0	0	26	20	0	0	0		
Percent ●	100	28	100	100	10	31	100	100	100		

Standardized assessment of study quality based on the Newcastle–Ottawa-Scale for cohort studies. Each of the 29 studies was assessed for the category’s selection (4 items), comparability (2 items), and outcome (3 items). Fulfilled and unfulfilled criteria are presented by of the solid rhomboid (●) and open circle (○), respectively. Study quality was graded as good (≥ 8 points), fair (6 or 7 points), and poor (≤ 5 points)

### Patient survival post KT

**Fig. 2:**
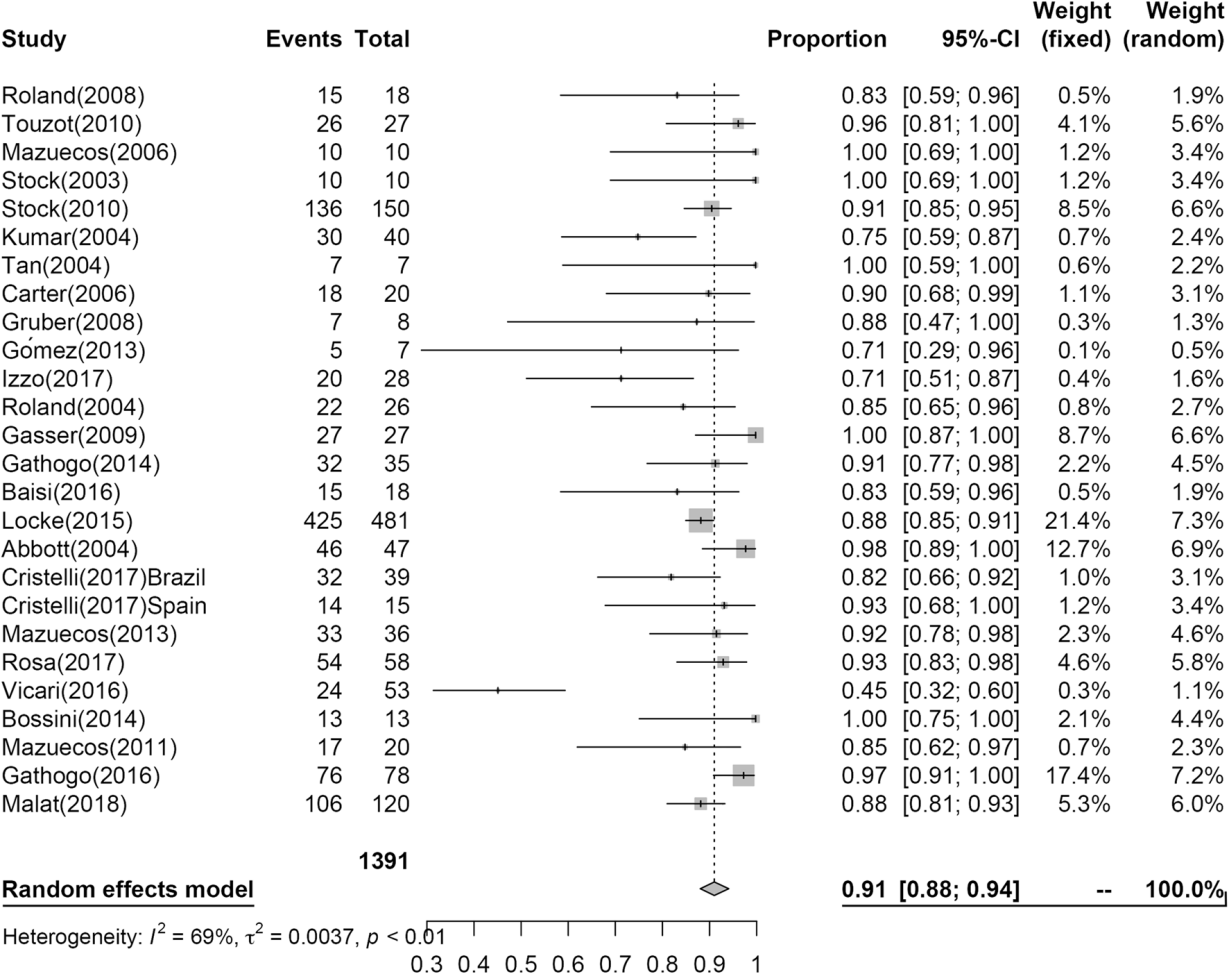
Pooled estimated proportion of patients surviving the first year, analyzed using a random effects model

**Fig. 3:**
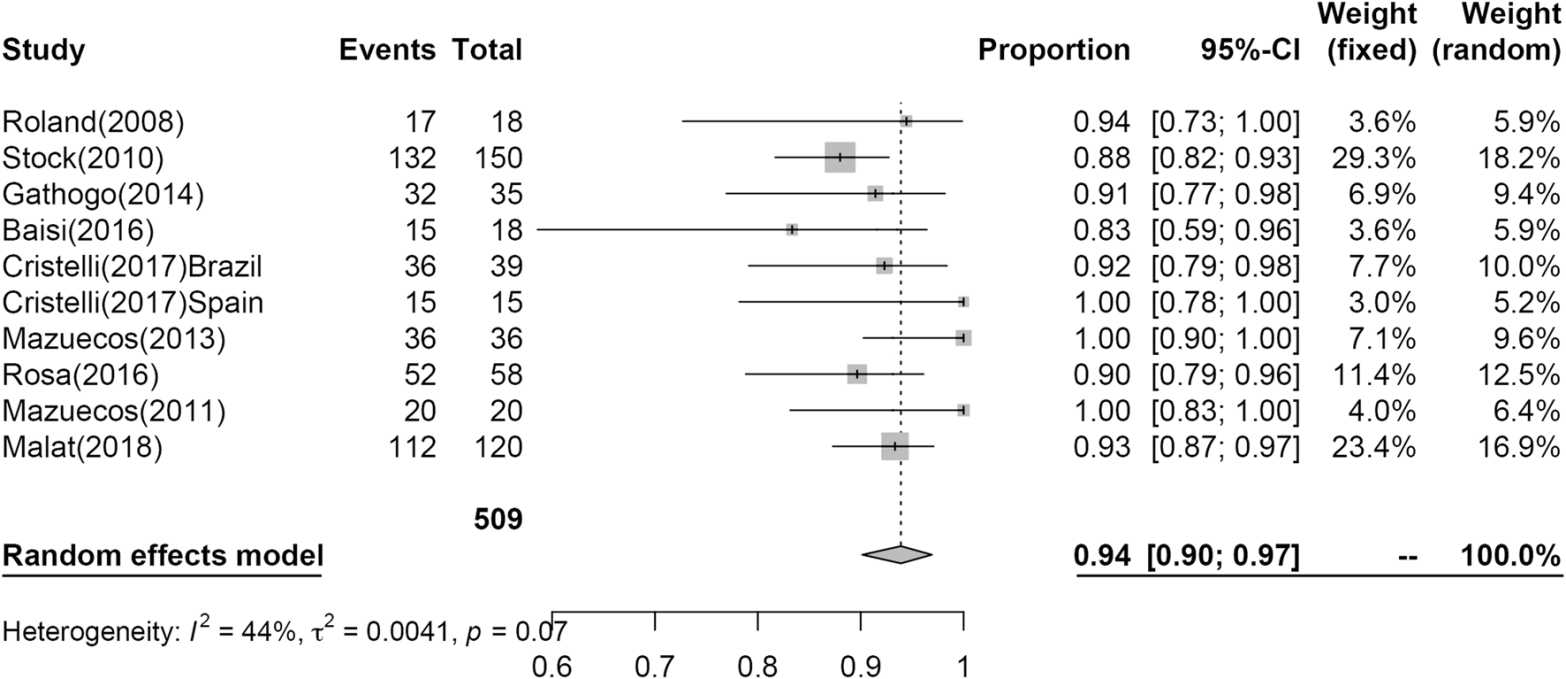
Pooled estimated proportion of patients surviving the third year, analyzed using a random effects model

Twenty-seven studies reporting PS at 1-year post KT included PS estimate to post KT for 1429 patients; however, only nine studies including 509 patients reported PS at 3 years. The results of the analysis are shown in Figs. 2 and 3. At 1 year, 97% (95% CI 0.95; 0.98, I^2^ = 36%) of patients survived, while 94% (95% CI 0.90; 0.97, I^2^ = 44%) of patients survived at 3 years.

### Graft survival post KT

**Fig. 4:**
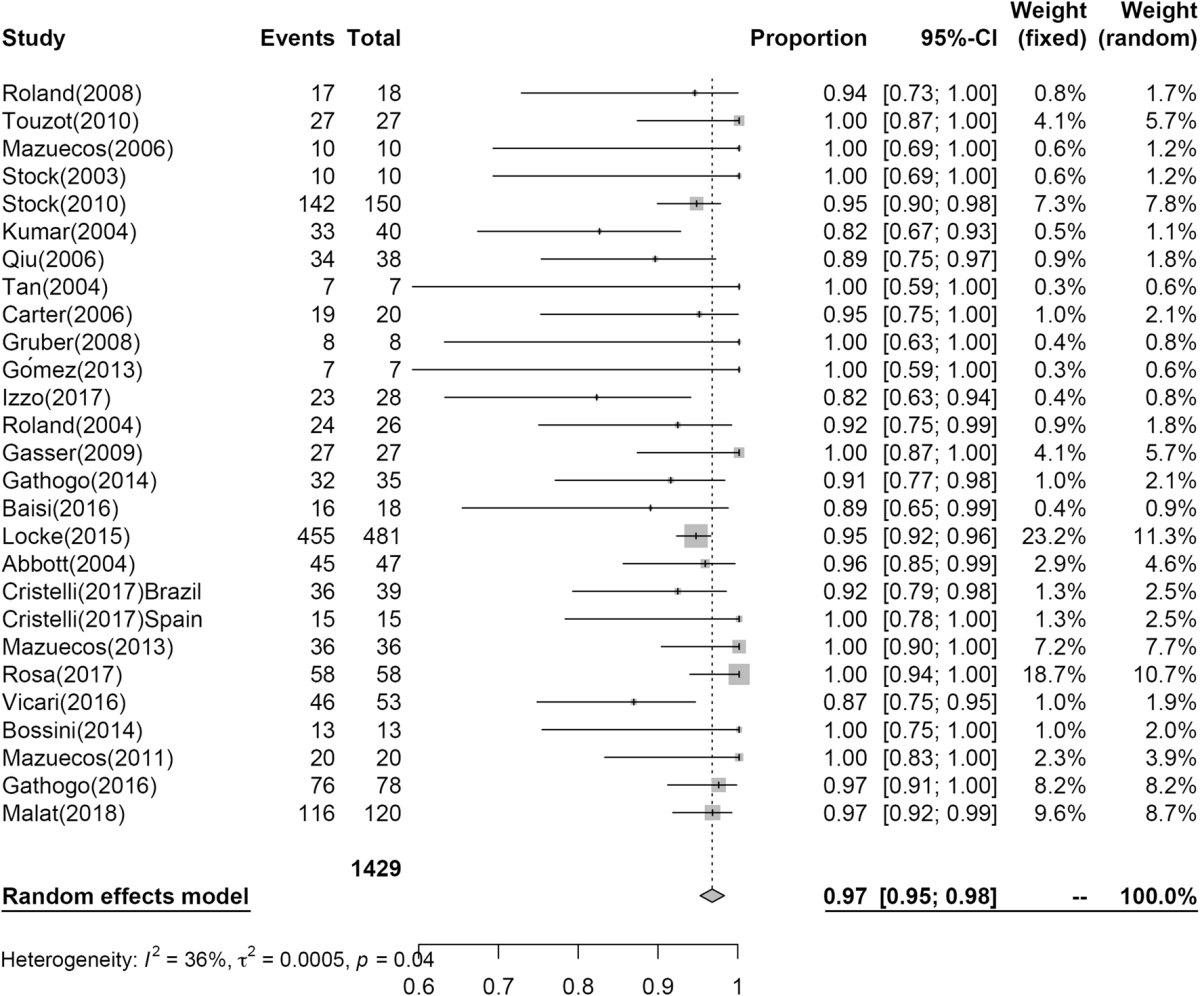
Pooled estimated proportion of graft surviving the first year, analyzed using a random effects model

**Fig. 5:**
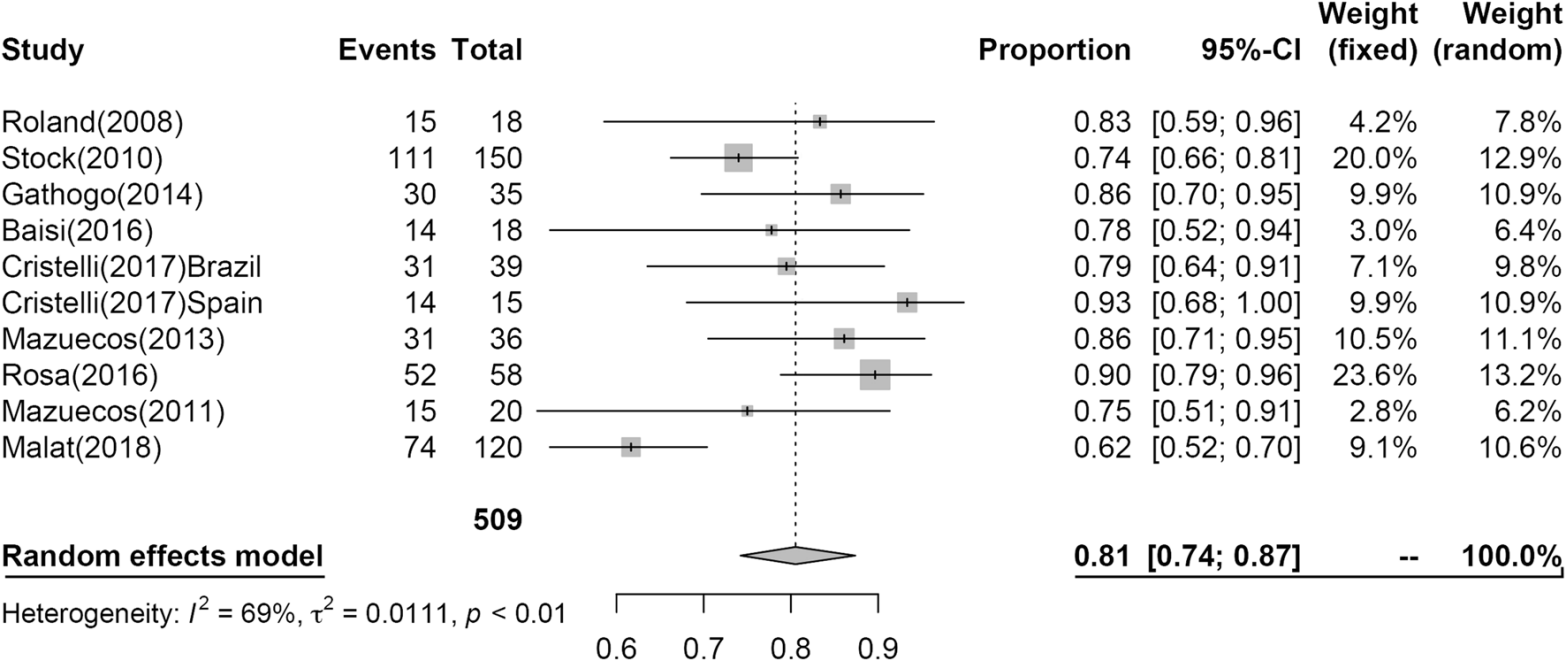
Pooled estimated proportion of graft surviving the third year, analyzed using a random effects model

Twenty-six studies including 1391 patients reported GS at 1-year post KT, and nine studies including 509 patients reported GS at 3 years. The results of the analysis are shown in Figs. 4 and 5. At 1 year, 91% (95% CI 0.88; 0.94, I^2^ = 69%) of grafts had survived, and GS subsequently declined to 0.81 (95% CI 0.74; 0.87, I^2^ = 69%) at 3 years.

### Acute rejection post KT

**Fig. 6:**
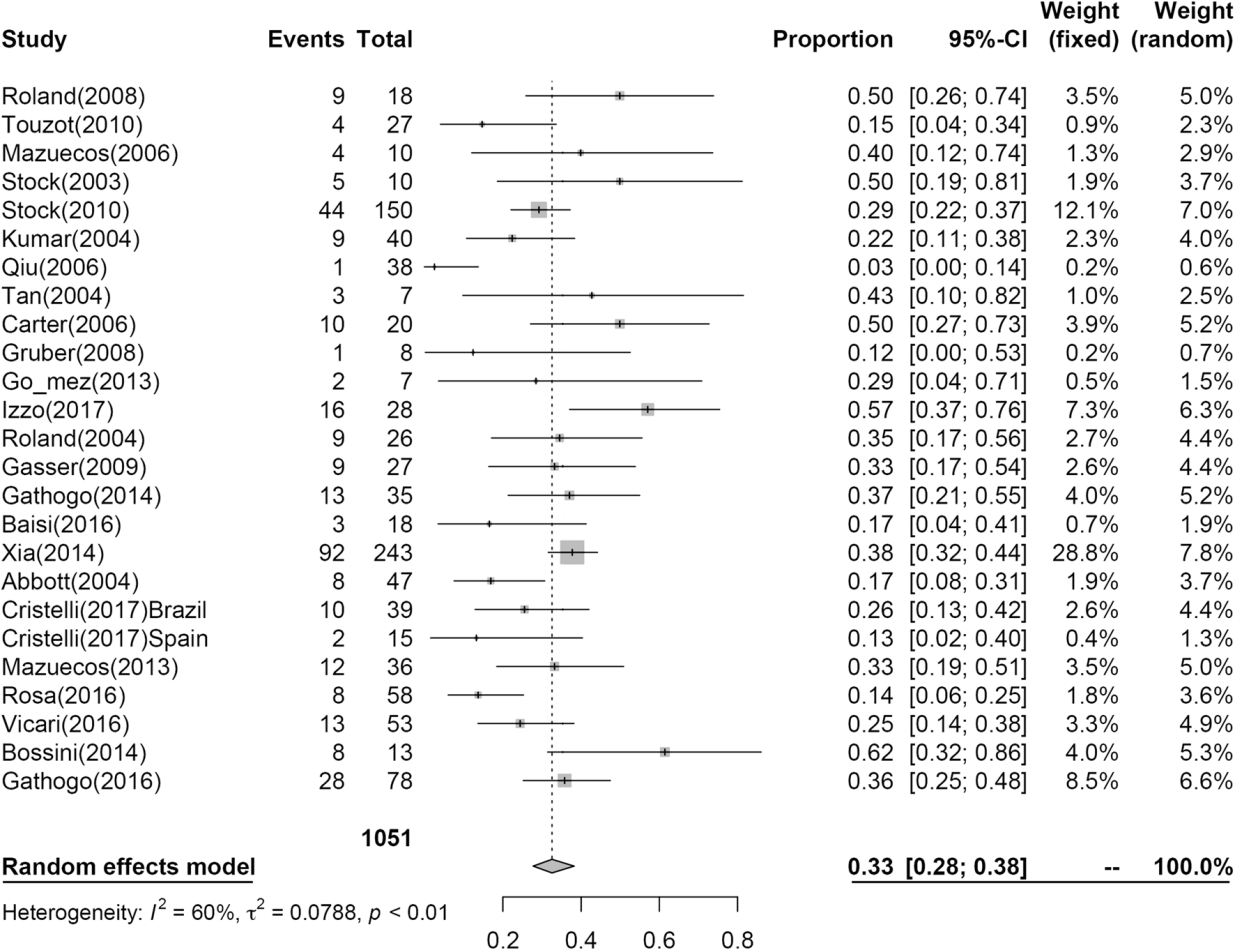
Pooled estimated proportion of acute rejection at the first year, analyzed using a random effects model

Twenty-five studies including 1051 patients reported AR post KT at 1 year, and the results of the analysis are shown in Fig. 6. At 1 year, 33% (95% CI 0.28; 0.38, I^2^ = 60%) of patients had AR.

### Infectious complications post KT

**Fig. 7:**
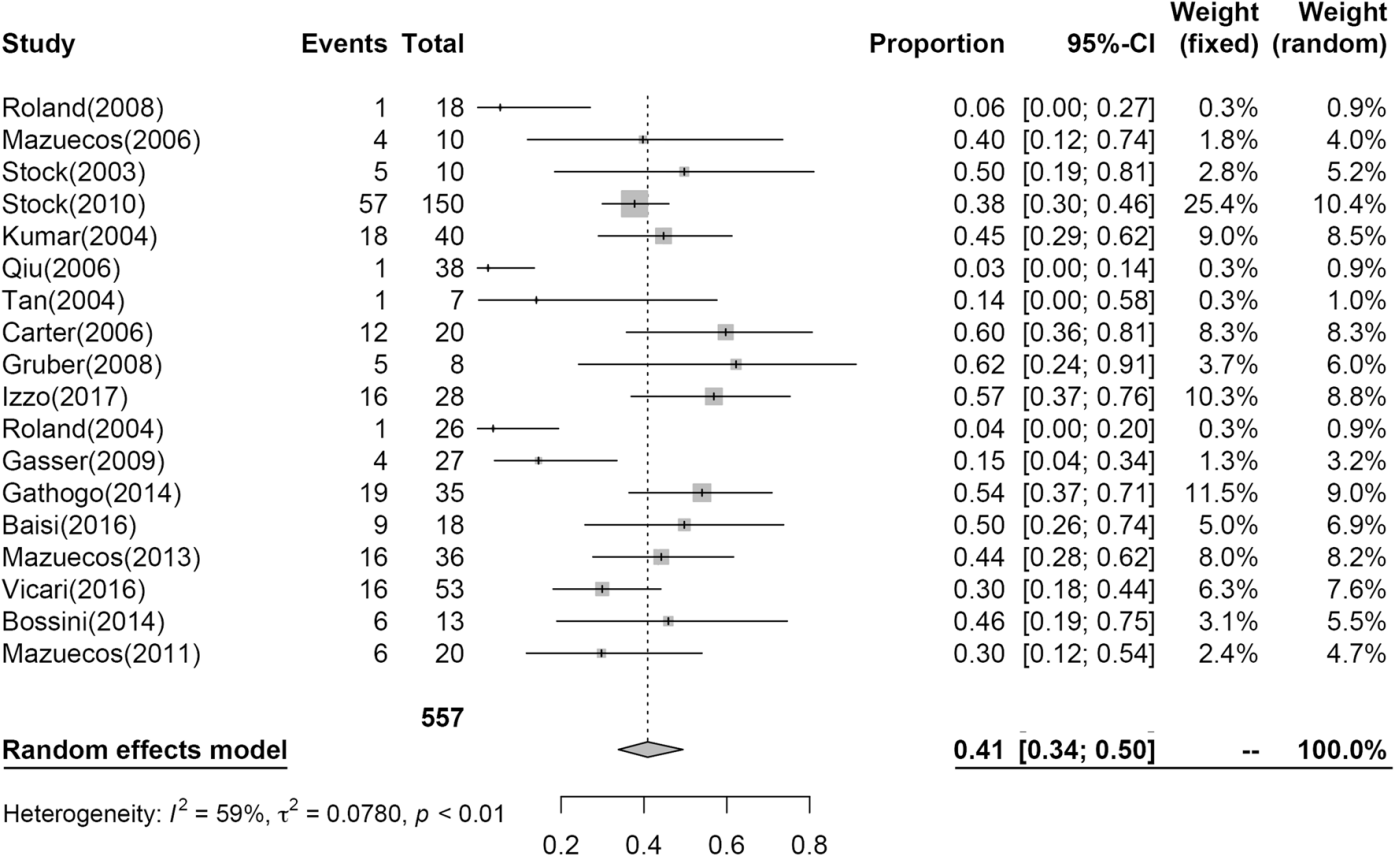
Pooled estimated proportion of infectious complication at the first year, analyzed using a random effects model

Nineteen studies including 584 patients reported IC post KT at 1 year; the results of the analysis are shown in Fig. 7. At 1 year, 41% (95% CI 0.34; 0.50, I^2^ = 59%) of patients had IC.

## Discussion

To our knowledge, this is the first systematic review and meta-analysis of such a large scale to report the outcomes of KT in HIV+ patients. We review and meta-analysis the outcomes in HIV+ KT patients, and looks at the 1- and 3-year GS/PS and AR rate.

The availability of cART has made KT a feasible treatment for selected HIV+ patients with ESRD, with outcomes somewhat inferior to those observed among the overall population of KT recipients [4, 9–11].

### Outcomes of KT

KT is now a viable treatment for select patients with HIV and ESRD. Moreover, the high incidence of morbidity and mortality resulting from cardiovascular issues in HIV+ patients [12, 13], as well as the negative effects of prolonged steroid use on conditions associated with cardiovascular risk, such as diabetes, dyslipidaemia, and hypertension, are well known [14, 15].

However, data regarding long-term outcomes and comparisons with appropriately matched HIV− patients are still lacking.

Locke et al. analysed 510 adult KT recipients with HIV matched 1:10 with HIV− controls. They found that HIV− and HIV mono-infected KT recipients had similar GS and PS, whereas HIV/HCV co-infected recipients had worse outcomes [16].

Izzo et al. found that the survival rate of patients was 82.1% and functioning grafts was 71.4% [17], and a recent report from the Italian national transplantation registry showed a PS rate of 95% and a GS rate of 85% between 2006 and 2014 [18].

Stock et al. [4] reported a survival rate of 94.6% 1 year after transplantation (88.2% after 3 years) in a multicentric trial (150 patients), and in a published review with a small number of patients, the survival rate was 93% within the first year of transplantation (254 patients) [19]. What’s more, as the high incidence of co-infection with HCV in HIV+ patients, co-infection is likely a driver of poor outcomes [20].

In our analyses, at 1 year, PS was 0.97 (95% CI 0.95; 0.98), GS was 0.91 (95% CI 0.88; 0.94), and at 3 years, PS was 0.94 (95% CI 0.90; 0.97), GS was 0.81 (95% CI 0.74; 0.87).

### Immunosuppression therapy

One of the most challenging goals in solid-organ transplantation is to tailor the immunosuppressive regimen for each individual patient to minimize immunosuppression while still preventing AR. Opportunistic infections and malignancies often attributed to immunosuppression itself remain a significant cause of death after transplantation. In the field of HIV+ organ transplantation, finding a balanced approach to immunosuppression is even more critical.

Currently, the vast majority of KT patients receive induction immunosuppression, which has been shown to greatly reduce the risk of rejection and improve PS and GS [21]. As shown in our analyses, most of the HIV+ KT patients received induction therapy. The two most commonly used induction agents are ATG and IL-2 receptor blocker (anti-IL2R) [22].

Guidelines from the Kidney Disease: Improving Global Outcomes transplant working group recommended anti-IL2R as the first-line treatment for patients at low risk for rejection and ATG for those at high risk [23].

Despite being the standard of care for most HIV patients, the use of induction immunosuppression for HIV+ patients, particularly ATG, remains controversial.

On the one hand, HIV+ patients have high rates of rejection and thus stand to benefit significantly from induction. On the other hand, the risks posed from prolonged lymphocyte depletion are of major concern given that HIV+ patients are perceived to already threaten T cell populations and reduced immunity, both states that are associated with an increased risk of opportunistic infections.

A recent study showed that ATG induction was associated with long-term impairment of T cell function and related infections, even after the patients had normalized CD4 counts [24]. This finding is also a reminder that the CD4 counts incompletely assesses the recovery of an immunocompetent CD4 T cell pool.

The incidence and severity of IC following transplantation are largely dictated by the recipient’s capacity for immune reconstitution. A study by Suarez et al. indicate that ATG-induced CD4 lymphopenia can be prolonged, and even at 1 year post transplant, a substantial proportion of patients has CD4 counts < 200/μL [25]. The baseline CD4 counts did not influence the risk of death, graft loss or AR. These findings suggest that although in current practice, HIV+ candidates with pre-transplant CD4 counts between 200 and 349/μL are eligible for KT [26] and are likely to have outcomes similar to those with higher counts, this group of patients carries a substantial risk of lymphopenia and associated infections following ATG induction.

A study by Bossini et al. showed that in HIV+ KT recipients treated with basiliximab and maintained on a calcineurin inhibitor (CNI)-mycophenolic acid (MPA)-based regimen, early corticosteroid withdrawal was associated with a very high incidence of AR and that kidney function was worse in patients with rejection [27].

However, in contrast to the studies above, in a large national cohort of 830 HIV+ KT recipients, Kucirka found wide variation in the use of induction immunosuppression, with > 30% of HIV+ patients receiving no induction compared with only 20% of their HIV counterparts. Therefore, the study indicated that the use of induction, including the lymphocyte-depleting agent ATG, was not associated with an increased risk of infections. Despite the fact that induction recipients were at higher risk for AR, the researchers observed lower rates of delayed graft function (DGF), AR, graft loss and days spent hospitalized in the first year after KT as well as a trend towards lower mortality. They suggested that the benefits of induction immunosuppression to prevent graft rejection in HIV+ KT recipients far outweigh the perceived risk of increased infections. Because the study had the largest sample size to date and the cohort was nationally representative rather than a select study population, this study claims to be more credible. Furthermore, the authors accounted for confounding and treatment selection bias, which previous studies did not do, and they did this using inverse probability of treatment weighting (IPTW), a method that allowed them to adjust for many clinical and demographic factors even when modelling relatively rare outcomes such as graft loss and death [28].

### Acute rejection

A high risk of AR is a well-known concern in HIV-infected kidney graft recipients. With regard to rejection, most studies observed a higher number of events in HIV+ patients than in HIV− patients. AR may occur as a result of immune dysregulation and the continuous inflammatory state of HIV+ recipients, in whom immunogenicity is increased following allograft implantation [4, 29].

Vicari et al. [30] evaluated the outcomes of KT in recipients with HIV infection under HAART in Brazil. The main results showed that HIV+ recipients presented a higher incidence of DGF, rejection, and bacterial infections and had lower PS and GS rates in comparison with a paired control group.

In the study, the incidence of treated AR was higher in the HIV+ group, and the incidence of biopsy-confirmed AR was numerically higher in this group. Additionally, even though an identical incidence of antibody-mediated AR occurred, the incidence of steroid-resistant rejection was numerically higher in the HIV group. Many reports have revealed an elevated incidence of AR in HIV+ recipients, varying between 31% and 55% [4, 9, 10], although a significantly lower incidence was reported in one study [31]. Stock et al. [4] reported that a significant proportion of acute cellular rejections were steroid resistant and that no episodes of antibody-mediated AR were observed in their cohort.

However, Malat et al. [32] described an elevated incidence of mixed cellular and antibody-mediated rejections. Furthermore, Locke et al. [33] reported that HIV+ patients who received ATG induction therapy had a much lower risk of rejection compared to patients without induction and that the risk was similar to uninfected controls.

Gathogo et al. [34] reported that TAC has an impact in reducing the incidence of AR in HIV+ recipients compared to cyclosporin A (CSA). The reasons for such an elevated incidence and severity of AR in HIV+ KT recipients are not clear. Dysregulation of the immune system along with a continuous inflammatory state caused by HIV infection, perhaps in association with a variability in drug exposure, has been hypothesized to explain these almost uniformly elevated incidences of rejection [4, 10, 35].

In addition, the elevated incidence of acute cellular rejection has been recently hypothesized to partially occur a result of an infiltration of inflammatory cells that occurs in response to tubular cell infection by HIV [36].

A study by Malat showed a relatively higher incidence of mixed rejection in HIV+ recipients compared with that reported for non-HIV transplant recipients. A donor terminal serum creatinine greater than 2.5 mg/dL predicted mixed rejection and was associated with poor outcomes. Donor selection and optimization of immunosuppression may be critical in these patients [36]. Even if rejection was controlled successfully with steroid therapy, these results, as previously reported, suggest a possible scenario where the immune system, damaged by HIV infection, has a worse response to immunosuppressive treatment with respect to the general population, even in patients without a severe immunological dysfunction at the time of transplantation. In our analyses, AR at 1 year was 0.33 (95% CI 0.28; 0.38).

### Infectious complications

During the first decades of the renal transplantation era, a serious IC developed in up to 70% of patients following transplantation, resulting in fatal outcomes in as many as 11% to 40% of cases [37]. In a recent case–control study with a median follow-up of 5 years, Ailioaie et al. [38] found a similar incidence of post-transplant IC in HIV+ KT recipients compared with matched KT HIV− controls. An IC incidence of 29% after transplantation was previously reported [19], and the incidence of post-transplant neoplasms has been described as similar to the incidence in HIV− patients. In our analyses, the incidence of IC observed at 1 year was 42% (95% CI 0.34; 0.50, I^2^ = 59%), and the rate of incidence of IC observed in this study in HIV+ KT patients is in line with the frequencies reported in a study by Stock et al. [4] where 38% of 150 HIV− KT recipients had at least one infection that required hospitalization.

However, the long-term patient and graft outcome of the whole cohort were not influenced by HIV status but were adversely influenced by infections, as survival was diminished in patients having at least one infection.

Furthermore, one-third of HIV+ KT recipients in a study by Ailioaie et al. did not have any episodes of infection, and repeated infections were not frequent. More importantly, the rate of incident infections was not different between the HIV+ and HIV− matched groups.

### Drug interaction

As experience with transplantation in HIV+ patients grow, significant drug–drug interactions between ART and maintenance immunosuppression have been identified as a major clinical challenge.

Post-transplant management of HIV infection with protease inhibitor (PI) and nonnucleoside reverse transcriptase inhibitor (NNRTI)-based ART is complicated by reciprocal drug interactions with immunosuppressive therapy, especially CNI, because of inhibition or induction of P450 cytochrome enzymes. Co-administration of PIs with CNIs requires significantly decreased CNI doses and prolonged dosing intervals to avoid supratherapeutic trough levels.

Despite appropriate CNI dose adjustments, variations of drug serum levels are difficult to control and have been linked to increased graft rejection in HIV+ KT recipients [34, 39].

In a study of 150 HIV+ KT patients, the largest to date, higher-than-expected rates of rejection were reported (31% and 41% at 1 and 3 years, respectively) [4]. The authors speculated that increased rates of rejection may have been secondary to altered CNI levels since only one-third of patients on PI- or NNRTI-based regimens underwent CNI dose adjustments.

In a study by Rosa et al., patients receiving PI-containing regimens had lower PS at 1 and 3 years than patients receiving PI-sparing regimens—85% vs. 100% (p = 0.06) and 82% vs. 100% (p = 0.03), respectively [40].

The increased risk of AR in HIV+ individuals has been largely attributed to reduced exposure to immunosuppressive agents due to drug–drug interactions with ART [4, 41, 42]. Other factors, such as infection of the allograft, previous alloimmunization and immune activation, might also play roles in predisposition to rejection [43].

This observation might be due to the effects of PI on tacrolimus levels, considering that the overwhelming majority of these patients were on a PI-containing regimen and that more than half had tacrolimus levels above target at the time of infection. PI could also influence the net state of immunosuppression by increasing the level or effect of other immunosuppressants, such as prednisone and mycophenolate.

The most important finding in the present study is the association between PI use and adverse outcomes, namely, reduced 3-year PS and GS, and increased risk of serious non-opportunistic infections. These observations remained true in analyses restricted to patients receiving nucleoside reverse transcriptase inhibitor (NRTI) “backbone”; thus, even after excluding the potential influence of other agents included in the ART regimen, PI continued to be associated with poor outcomes.

However, the use of NNRTI or tenofovir disoproxil fumarate (TDF) did not influence GS. Tenofovir alafenamide (TAF) is a new formulation of tenofovir associated with less kidney (and bone) toxicity [44]. Whether TAF has added clinical benefit over TDF in KT recipients remains to be established.

In a large single-centre study of HIV+ KT recipients conducted by Boyle et al. [45], treatment with TDF at the time of transplant was not associated with 36-month death-censored primary allograft loss after adjustment for DGF and a propensity score for TDF exposure.

Given that specific recipient characteristics, such as hepatitis B co-infection and certain HIV mutations, continue to make TDF-based regimens the most likely to provide adequate viral suppression post-transplant, despite observational data for nephrotoxicity in the non-transplant population.

However, given the limitations of this study, TDF should be reserved for patients who have limited ART options and should be used very cautiously in the KT population, with appropriate dose adjustment and surveillance of kidney function, including kidney biopsy when indicated. Substituting TAF for TDF in KT patients is reasonable, but it should be noted that no data are yet available on long-term kidney outcomes with TAF in KT and non-KT recipients in the setting of both preserved and reduced glomerular filtration rate (GFR).

Since their introduction in 2007, integrase strand transfer inhibitors (INSTIs) have been proposed as preferred post-transplant ART because of a favourable pharmacologic profile with decreased potential for drug interactions [3, 4, 42, 46]. In a study by Stock et al. [4] the majority of patients were on PIs or NNRTIs with only 4% of participants receiving INSTIs; these patients were also receiving PI, NNRTI or maraviroc, making it impossible to draw conclusions about INSTI-based therapy. In a series of 27 HIV+ KT patients in France predominantly on PI or NNRTI-based regimens (93%), 70% required post-transplant ART modification due to drug interactions with CNIs [32].

Recently, Alfano et al., reported that preferred drug included raltegravir and dolutegravir for INSTI class, maraviroc for CCR5 receptor antagonist, lamivudine for NRTI, and rilpivirine for NNRTI, which offered advantage of having no drug interactions [47].

In summary, we believe that INSTI or CCR5-based therapy should be the preferred ART in patients with HIV who undergo KT, primarily because of decreased drug–drug interactions with immunosuppressive medications such as CNIs, enabling easier monitoring of immunosuppressive medications and superior graft outcomes. However, larger and more controlled trials are needed to better assess the long-term outcomes of INSTI-based therapy to elucidate factors related to GS other than direct reciprocal drug interactions.

## Conclusions

In conclusion, this systematic review and meta-analysis demonstrated that with careful selection of patients and multidisciplinary evaluation, KT can be performed with good outcome in HIV+ patients. Moreover, with the advent of INSTI-based cART regimens, drug–drug interactions between cART and immunosuppressants have been dramatically reduced. Nevertheless, further studies are needed to optimize immunosuppressive therapy regimens for HIV+ patients, with the aim of reducing the high rate of AR after transplantation. Furthermore, this review still has its limitations, such as lack of sufficient studies, possibility of some overlapping patient cohorts, short of comparator. And we are also looking forward to other novel papers as more and more studies regarding KT of HIV+ patients.

## Data Availability

The datasets during and/or analysed during the current study available from the corresponding author on reasonable request.

## Abbreviations

HIV: human immunodeficiency virus
AIDS: Acquired Immune Deficiency Syndrome
HAART: highly active antiretroviral therapy
KT: kidney transplantation
cART: antiretroviral combination therapy
ESRD: end-stage renal disease
CD4 counts: CD4 T cell counts
AR: acute rejection
PS: patient survival
GS: graft survival
IC: infectious complications
PRISMA: Preferred Reporting Items for Systematic Reviews and Meta-Analysis
NOS: Newcastle–Ottawa-Scale
ATG: antithymocyte globulin
CSA: cyclosporin A
MMF: mycophenolate mofetil
TAC: tacrolimus
MPA: mycophenolic acid
PI: protease inhibitor
DGF: delayed graft function
IPTW: inverse probability of treatment weighting
NNRTI: nonnucleoside reverse transcriptase inhibitor
